# Maternal health service utilization in the Jimma Zone, Ethiopia: results from a baseline study for mobile phone messaging interventions

**DOI:** 10.1186/s12884-024-06683-w

**Published:** 2024-07-17

**Authors:** Gebeyehu Bulcha, Hordofa Gutema, Demisew Amenu, Zewdie Birhanu

**Affiliations:** 1 Department of Health, Behavior, and Society, Faculty of Public Health, Institutes of Health, Jimma University, Jimma, Ethiopia; 2 Department of Maternal, Newborn and Child Health, Oromia Regional State Health Bureau, Jimma Zone Health Office, Jimma, Ethiopia; 3 Department of Obstetrics and Gynecology, Faculty of Medical Sciences, Institutes of Health, Jimma University, Jimma, Ethiopia

**Keywords:** Maternal health, mHealth, Determinants, Jimma, Ethiopia

## Abstract

**Background:**

Over the last 20 years, global healthcare has placed a major focus on improving the survival rates of mothers and children, recognizing the potential to prevent a significant number of deaths resulting from pregnancy and childbirth. Nevertheless, there remains an ongoing obstacle to the insufficient utilization of critical obstetric services to achieve optimal health outcomes for pregnant women. This study aimed to assess the magnitude and determinants of maternal healthcare use in the Jimma Zone, Ethiopia.

**Methods:**

Data were obtained from a household survey as part of the baseline assessment of a cluster randomized controlled trial. The study participants comprised 588 women in early pregnancy, specifically those with a gestational age of less than 20 weeks. Logistic regression analysis was employed to identify factors associated with the use of maternal health services. Adjusted odds ratios (AORs) were used to assess the strength of the associations, with significance level set at a *p*-value ≤ 0.05.

**Results:**

The overall prevalence of maternal health service utilization was 87.9% (CI: 85.1, 90.4) for antenatal care, 74.7% (CI: 73.2, 79.9) for health facility delivery, and 60.4% (CI: 56.4, 64.3) for postnatal care. Multivariable logistic analysis revealed that maternal health service use was significantly influenced by access to health facilities (AOR: 6.6; 95% CI: 2.39, 18.16), financial hardship (AOR: 3; 95% CI: 1.97, 4.61), perceived respectful care (AOR: 2.3; 95% CI: 1.07, 5.11), perceived privacy of service provisions (AOR: 2.4; 95% CI: 1.47, 4.06), and attitudes toward maternal service use (AOR: 2.2; 95% CI: 1.48, 3.24).

**Conclusions:**

The study revealed slightly higher rates of antenatal care, facility delivery, and postpartum care utilization. However, there was a low proportion of early antenatal care initiation, and high rates of antenatal care dropout. Mobile phone-based messaging intervention may enhance maternal health service use by addressing factors such as lack of access, economic challenges, disrespectful care, no privacy of procedures, and unfavorable attitudes.

## Background

Maternal health is a pillar within the broader landscape of global health concerns. It serves as an essential indicator that shapes the overall health and developmental trajectory of a society [1, 2]. Most maternal deaths worldwide occur in sub-Saharan Africa, with 70% of the burden in this region. The estimated maternal mortality ratio (MMR) is 542 deaths per 100,000 live births, which exceeds the target set in the Sustainable Development Goal (SDG) of 70 per 100,000 live births by 2030 [3–6].

Reducing maternal mortality is critical to achieving Sustainable Development Goal 3 (SDG_3_), which aims to ensure good health and well-being for people of all ages [7]. Experts predict that the target of reducing maternal mortality as part of the SDGs can be met by ensuring that 91% of women receive their first antenatal care visit (ANC1), 78% receive their fourth antenatal care visit (ANC4), eighty-one percent opt for institutional delivery, and 87% have skilled birth attendance by 2030 [5, 8–10]. Despite remarkable efforts, maternal death rates continue to be high in settings with limited resources. Ethiopia has one of the most alarming rates of maternal mortality worldwide [11, 12].

Although the effective use of maternal health services is a proven strategy to reduce maternal mortality, the utilization of maternal and neonatal health services and the level of health practices are very limited in many developing regions of the world [13]. According to the 2019 Ethiopia Demographic and Health Survey (EDHS), 74% of women receive antenatal care from skilled providers, 51% of births occur in health facilities, 38.5% receive postnatal check-ups, and 34% have four or more antenatal care visits [11].

There have been reports of discrepancies in local access to vital maternal health services. For instance, a study conducted by Mussie Alemayehu in the Tigray region showed that 58.2% of women attended antenatal care 4^+^, 87.9% opted for institutional delivery, and only 40.3% received postnatal care within 42 days of giving birth [14]. A study conducted in Ambo town, Oromia Regional State, revealed that the utilization of maternal health services was 39.7% for antenatal care, 28.7% for delivery services, and 19% for postnatal care [15]. Similarly, a study in the Amhara region revealed a low prevalence of maternal healthcare service utilization, with only 77% of women receiving Antenatal care (ANC) and 37% receiving postnatal care [16].

Multilevel factors, including sociocultural beliefs and practices, socioeconomic conditions, access to healthcare services, maternal education, lengthy wait times at healthcare facilities, inadequate referral procedures, poor staff interpersonal interactions, lack of privacy, and mothers' prior unfavorable experiences with the health system [17–22], influence the uptake of key maternal health services.

Research has shown a correlation between household wealth [23], health insurance [24], and maternal health service use. Education is an individual-level factor that consistently predicts service maternal health service uptake [25]. Middle-aged women are more likely to visit healthcare facilities during childbirth [26], and women's occupations, knowledge, and attitudes significantly influence maternal care practices [27, 28]. Inadequate social support prevents women from making appointments with healthcare providers [29]. Women with strong superstitious beliefs are less likely to utilize all three types of maternal health care [30]. Cultural beliefs and practices also affect care-seeking behavior, with women having limited rights to make decisions and limited time available to them [31]. Negative perceptions and poor attitudes discourage women from using intended services [32]. The extent of time available to women is also significant; in less-developed countries, more than two-thirds of women spend time caring for children and fetching water or fuel, leaving hardly any time for their health [33]. This study aimed to assess the magnitude of maternal health service use and associated factors.

## Methods and materials

### Study setting and design

We conducted a household survey as part of the baseline assessment of a cluster randomized controlled trial in three selected districts of the Jimma Zone, located 352 km from the capital, Addis Ababa in southwest Ethiopia. According to the projection of the 2007 population census, the three districts have an estimated population of 624,534 (49.32% women) by 2024. Of the total population, 144,267 (23.1%) were women of childbearing age. In the three districts, there are a total of 9 small urban and 75 rural gandas (lowest administrative units). The study was conducted from March 5 to March 20, 2023, as part of the baseline assessment in an ongoing cluster randomized controlled trial (cRCT). The trial protocol has been accepted for publication [34] and the trial was registered on January 4, 2022 with Clinical Trials (trial identifier PACTR202201753436676) and can be accessed at https://pactr.samrc.ac.za.

### Study population

We considered Gandas with functional health posts and mobile network coverage as inclusion criteria for this study. However, throughout the selection process, almost all health posts were functional, had access to mobile networks, and were eligible for selection. The study population included pregnant women in the selected ganda of the three districts. A woman was included in the study if her Gestational Age (GA) was less than 20 weeks, as determined by the midwives through a thorough house-to-house registration process using the last menstrual period (LMP) and/or fundal height measurement technique. In Ethiopia, for frontline health facilities like health posts and health centers, gestational age (GA) is primarily determined using the last menstrual period (LMP). When women are unable to recall their LMP, health professionals use the fundal height measurement technique based on guidelines from the Federal Ministry of Health. Although these techniques are less accurate than ultrasound, they serve as primary methods for GA estimation in frontline health facilities because of their practicality and feasibility. In addition, the participant was included if she or one of her family members owned a mobile phone and provided written informed consent showing a willingness to participate in the study. Informed consent was obtained only from those randomly selected participants. The study excluded women who fulfilled the enrollment criteria but were critically ill at the time of enrollment and women with psychological disorder.

### Sample size determination

We used methods proposed by Hooper and Bourke for sample size calculation [35] assuming a simple parallel group with a baseline outcome assessment and one follow-up assessment. Three trial arms (Gain framed, loss-framed, and the control arms) were assumed. The sample size, assuming individual randomization, was calculated with the following assumptions: for P1, the proportion of women attending ANC4 + among the control group was 23.27% [36], the power (1-β) was set at 0.8, and the two-sided alpha was 0.025. The effect size/absolute difference in the proportions of antenatal care visits > 4 was 0.15, and Zα/2 was adjusted for multiple comparisons using a Bonferroni correction = 2.24. The initial sample size (no) was calculated as follows: [no = DE* (Zα/2 + Zβ)2 * (P1(1 − P1) + P2(1 − P2))/(P1 − P2)2, where no was 175. Two design effects were used to inflate the sample size under individual randomization, considering the within-period intracluster correlation coefficient (ICC) and the between-period ICC. The design effect (dc) due to cluster randomization was calculated by considering the following parameters: within-period ICC (p) of 0.003 [37] and number of clusters per arm (n × dc × dr) ÷ m = 7. We calculated the design effect as dc = 1 + (m − 1)ρ = 1.12. The design effect (dr) due to repeated assessments was calculated using the following assumptions: within-period ICC and a cluster (0.003), autocorrelation coefficient (π) of 0.8 to allow for a 20% decay of the correlation from within to between different periods [38], and correlation between sample means from the same cluster at different times (r) determined as r = mpΠ/1 + (m-1)p. The design effect due to repeated assessment was calculated as dr = (1-r2) = 0.996. To obtain the final sample size per arm, we multiplied the sample size for individual randomization with both design effects as (no × dc × dr) = 188. Considering a 4% loss to follow-up, the final sample size for each arm was 196. This resulted in twenty-eight women per health post for a total sample size of five hundred eighty-eight.

### Sampling technique

**Fig. 1:**
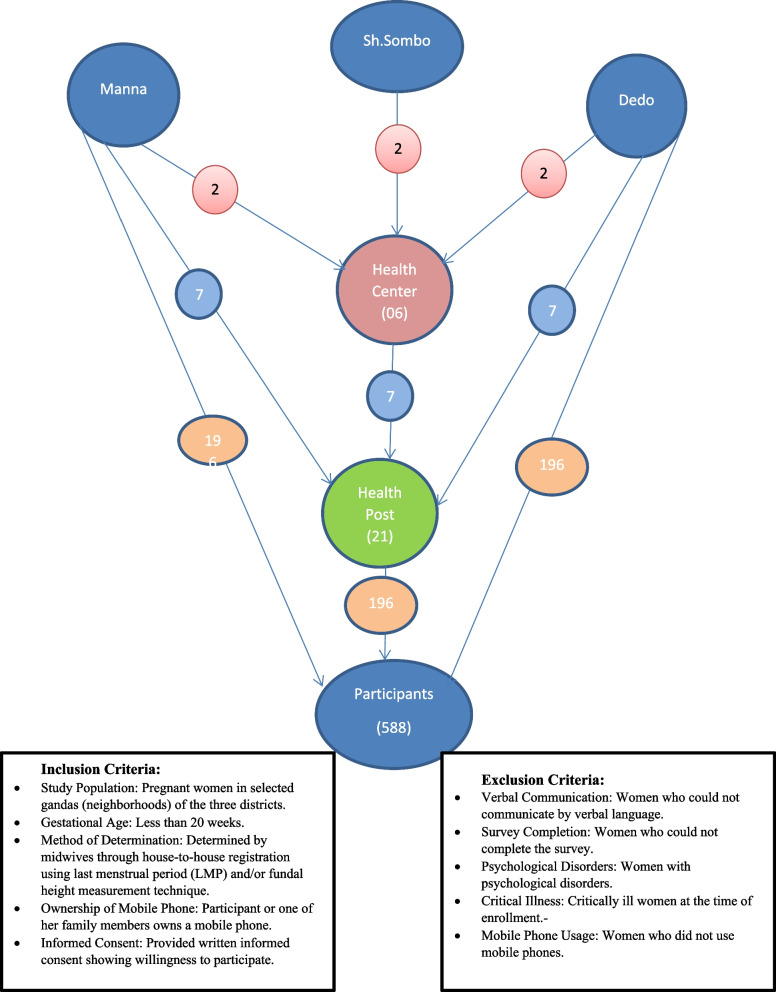
Cluster RCT with Elective Districts

We randomly selected two primary healthcare units (PHCUs) per district from the three districts identified for mobile phone-based messaging interventions. Seven health posts per district were randomly selected for the trial using an Excel random number generator. A simple random sampling technique was used to select pregnant women. An individual who was not involved in the implementation of the trial used an Excel number generator to allocate pregnant women to the three intervention arms (Fig. 1).

### Measurements

A structured questionnaire was used to measure maternal health service uptake and associated factors. The questionnaire was developed based on a review of the literature and presented in Afan Oromo language. The internal consistency of the key constructs was checked using Cronbach’s alpha, and all constructs showed acceptable Cronbach alpha coefficient values. The Cronbach’s alpha coefficient scores for knowledge, attitude, and self-efficacy were 0.81, 0.92, and 0.86 respectively. The outcome of the study was maternal health service utilization practices, which included giving birth in a health facility, making eight ANC follow-up appointments, and attending Postnatal Care (PNC) follow-up seven days after giving birth [39].

The study assessed knowledge of maternal health care practices using 16 questions covering knowledge related to pregnancy, labor, delivery, and postpartum periods, with correct answers indicating greater understanding and a higher score indicating greater knowledge when the correct answers' scores were combined. Attitude toward maternal care practice was measured by 10 items with a 5-point Likert scale ranging from 1 for strongly disagree to 5 for strongly agree. The cumulative scores were used in the analysis once negatively phrased items were reverse-coded. A higher composite score indicated a more favorable attitude toward maternal care.

Self-efficacy in practicing maternal care refers to an individual`s belief in their ability to perform maternal care practices. It was measured by 10 items designed on a 5-point Likert scale ranging from 1 for strongly disagree to 5 for strongly agree. Once negatively worded items were reverse-coded, the sum score was used in the analysis. A higher score indicated greater self-efficacy in practicing maternal care.

The household wealth level was calculated using principal component analysis, taking into account 15 household asset properties and categorizing the household wealth index into wealth quintiles (lowest, second, middle, fourth, and highest).

### Data collection methods

The data were collected via an interviewer-administered questionnaire by 21 (seven per district) data collectors who held at least a bachelor’s degree, were nursing school graduates with working experience in obstetric care, and were trained for three days. Each interview lasted for 45 min and was conducted based on the woman's preference. The data were checked for inconsistencies and errors during the data collection period by dedicated supervisors. To ensure accuracy and consistency, data collectors and supervisors received extensive preparatory training.

### Data processing and analysis

The data were checked, cleaned, and entered into Epidata version 4.6 (released in 2020, Denmark) and exported to SPSS version 25 (released in 2017, USA) for analysis. The Hosmer and Lemeshow test was used to examine the model's goodness of fit; *p*-values greater than 0.05 indicated a good fit. We used descriptive statistics to summarize the data. Bivariate analysis was performed, and variables with *p* values less than 0.25 identified as candidates for inclusion in the multivariable logistic regression. A multivariable logistic regression model was used to identify the associations of independent variables with the study outcomes. A 95% confidence interval and *p*-value less than 0.05 were considered to indicate a statistically significant association.

## Results

### Demographic characteristics

The study included 588 women with a gestational age of 16–20 weeks (100% response rate). The individuals' background characteristics are shown in Table 1. Over three-quarters (85.4%) of the participants were between the ages of 20 and 34, and 66.2% resided in rural areas. Regarding occupations; 67.9% of the pregnant women were unemployed, while 85.4$ of their male partners were self-employed. The wealth distribution skewed toward the medium category, comprising 80.4% of the surveyed population.

**Table 1:** Sociodemographic characteristics of the respondents (pregnant mothers) from the Jimma Zone, Oromia Regional State, Southwest Ethiopia (*n* = 588)

Variables	Categories	Number (%)
Age (year)	< = 20 Yrs	46(7.8)
	20 -34 Yrs	502(85.4)
	35—44 Yrs	40(6.8)
Residence	Rural	389(66.2)
	Urban	199(33.8)
Ethnic group	Oromo	557(94.7)
	Amhara	12(2)
	Others^a^	19(3.2)
Religion	Muslim	539(91.7)
	Others^b^	49(8.3)
Marital status	Married	553(94)
	Not in marriage	35(6)
Education level of Pregnant Women	No formal education	137(23.3)
	Can read and write	23(3.9)
	Grade 1to4	151(25.7)
	Grade 5to8	176(29.9)
	Grade 9 to12	72(12.2)
	College and above	29(4.9)
Education level of male partners	No formal education	75(12.8)
	Can read and write	23(3.9)
	Grade 1to 4	165(28.1)
	Grade 5to8	184(31.3)
	Grade 9 to12	103(17.5)
	College and above	38(6.5)
Occupation (pregnant women)	Self-employed^c^	151(25.7)
	Employed^d^	29(5)
	Unemployed^e^	408(69.3)
Occupation (male partners)	Self-employed^f^	151(25.7)
	Employed^g^	29(10.2)
	Unemployed^h^	26(4.4)
Wealth category	Poorest	6(1)
	Poor	54(9.2)
	Medium	473(80.4)
	Rich	55(9.4)

^a^(5 Yem, 5 Kefa, 8 Dawuro and 1 Silte)

^b^(30 Orthodox, 17 Protestant, and 2 Catholic)

^c^(118 farmer and 33 Merchant)

^d^(18 government employees, 11 daily laborers)

^e^(399 housewife, & 9 studens)

^f^(434 farmer and 68 Merchant)

^g^(30 government employees, 1 private organization employee, and 30 daily laborers)

^h^(13 retired & 13 students)

### Access factors and knowledge, attitudes, and self-efficacy (KASE)

Only 6.3% of the participants reported having immediate access to health facilities, whereas 30.8% reported moderate access (15–30 min). Additionally, 40.1% reported having reasonable access (30–60 min), and the remaining participants reported limited (60–90 min) and challenging (90 + minutes) access to health facilities.

Overall, for 59.2% of the women, walking was the primary mode of transportation to reach healthcare facilities; however, 31.1% had to pay for transportation. The majority (82.7%) of respondents had community-based health insurance. Most participants (93.2%) shared their opinions about not receiving respectful treatment, and approximately three-fourths (83.5%) reported having privacy when receiving services. However, 41% of respondents reported lengthy wait times. Only 42% of the respondents expressed a favorable attitude toward maternal health care.

Concerning the study participants' knowledge levels, 63.8% lacked adequate knowledge about maternal health care, and nearly half (53.1%) showed a high level of self-efficacy toward maternal health care.

A total of 6.1% of those in the gain frame, 4.6% in the loss frame, and 8.2% in the control arm had immediate access to healthcare facilities. Regarding the pattern of transportation, walking was widely relied upon by all groups. The control group had higher expenditures for transportation (41.3%) and healthcare services (53.6%). The gain-framed arm had a higher percentage of members with community-based health insurance (86.7%). Perceived respectful care was high (93.9%) among the gain-framed arms. More privacy experiences were found in the control group (92.9%). A greater percentage of participants in the loss-framed arm reported short waiting times (66.8%), whereas 51.5% of participants in the control arm experienced long waiting times (Table 2).

**Table 2:** Descriptions of access factors and knowledge, attitude, and self-efficacy (KASE), Jimma Zone, Oromia Regional State, Southwest Ethiopia (*n* = 588)

Variables	Categories	Gain framed n (%)	Loss framed n(%)	Control n(%)	Total n(%)	Statistical test (*P*.Value)
Access to the health facility^a^	Immediate Access	28(14.3)	25(12.8)	6(3.1)	59(10)	χ2 = 65.27 *p* < 0.001
	Moderate Access	41(20.9)	66(33.7)	74(37.8)	181(30.8)	
	Reasonable Access	66(33.7)	80(40.8)	90(45.9)	236(40.1)	
	Limited Access	49(25)	16(8.2)	10(5.1)	75(12.8)	
	Challenging Access	12(6.1)	9(4.6)	16(8.2)	37(6.3)	
Transportation mode	Ambulance	2(1)	19(9.7)	41(20.9)	62(10.5)	χ2 = 84.21 *p* < 0.001
	Public transport	41(20.9)	30(15.3)	45(23)	116(19.7)	
	Motorcycle	24(12.2)	4(2)	32(16.3)	60(10.2)	
	Walking	129(65.8)	141(71.9)	78(39.8)	348(59.2)	
	Others	0(0)	2(1)	0(0)	2(0.3)	
Economic hardship	Yes	66(33.7)	36(18.4)	81(41.3)	183(31.1)	χ2 = 24.66 *p* < 0.001
	No	130(66.3)	160(81.6)	115(58.7)	405(68.9)	
Member of CBHI^b^	Yes	170(86.7)	166(84.7)	150(76.5)	486(82.7)	χ2 = 7.97 *p* = 0.019
	No	26(13.3)	30(15.3)	46(23.5)	102(17.3)	
Pay for services	Yes	52(26.5)	49(25)	105(53.6)	206(35)	χ2 = 44.49 *p* < 0.001
	No	144(73.5)	147(75)	91(46.4)	382(65)	
Perceived respectful care	Yes	12(6.1)	10(5.1)	18(9.2)	40(6.8)	χ2 = 2.79 *p* = 0.248
	No	184(93.3)	186(94.9)	178(90.8)	548(93.2)	
Privacy	Yes	40(20.4)	43(21.9)	14(7.1)	97(16.5)	χ2 = 18.84 *p* < 0.001
	No	156(79.6)	153(78.1)	182(92.9)	491(83.5)	
Waiting time	Short waiting time	121(61.7)	131(66.8)	95(48.5)	347(59)	χ2 = 14.57 *p* = 0.001
	Long waiting time	75(38.3)	65(33.2)	101(51.5)	241(41)	
Knowledge	Knowledgeable	61(31.1)	20(10.2)	132(67.3)	213(36.2)	χ2 = 141.83 *p* < 0.001
	Less knowledgeable	135(68.9)	176(89.8)	64(32.7)	375(63.8)	
Attitude	Favorable attitude	30(15.3)	75(38.3)	142(72.4)	247(42)	χ2 = 133.05 *p* < 0.001
	Unfavorable attitude	166(84.7)	121(61.7)	54(27.6)	341(58)	
Self-Efficacy	Low Self-Efficacy	84(42.6)	92(46.9)	100(51)	276(46.9)	χ2 = 2.62 *p* = 0.270
	High Self-Efficacy	112(57.1)	104(53.1)	96(49)	312(53.1)	

^a^Immediate access (0–15 min), moderate access (15–30 min), reasonable access (30–60 min), limited access (60–90 min), and challenging access (90 + minutes)

^b^*CBHI* Community Based Health Insurance

### Literacy in using mobile phones

This relevant finding was from an accepted manuscript entitled `pregnant mother’s intention to use mobile phone-based messaging interventions for improving maternal and newborn health practices in Jimma Zone, Ethiopia’ which was part of cRCT. The study indicates a high level of literacy in mobile phone usage among pregnant women in Jimma Zone, Ethiopia. A substantial majority (79.9%) of the participants demonstrated the ability to read, with about half (54.1%) capable of sending mobile phone text messages. The majority (69.5%) of respondents had been using a mobile phone for 1 to 4 years. More than three-fourths (77.4%) of the respondents stated that a family member shares information they receive through their phone. Furthermore, nearly all respondents (98.9%) expressed a preference for receiving health information in Afan Oromo via text messages [40].

### Experience in prenatal care

The majority (95.1%) of the study participants were multigravida. Regarding pregnancy outcomes, 93.4% of the pregnant mothers gave birth to live children, 3.6% to stillbirths, 2.0% to miscarriages, and 1.0% to abortions. The prevalence of antenatal care (ANC) attendance was 87.9% CI (73.2, 79.9). Concerning early ANC initiation, more than three-quarters (77.4%) of the women sought healthcare before 16 weeks. A limited frequency of ANC (≤ 4 times) was reported by the majority (88.2%) of the research participants.

Regarding counseling during ANC, 70% of respondents reported receiving information about delivery preparation, 63.4% about breastfeeding, 56.3% about spacing children, 61.9% about immunization, 60.0% about danger signs of pregnancy, and 58.8% about nutrition. The majority (82%) reported having received a tetanus toxoid (TT) vaccination. Only 31.5% of participants stated that they always use insecticide-treated nets (ITNs), 23.3% reported using them occasionally, 12.2% seldom, and 33.0% never.

Regarding the range of services offered to pregnant mothers during routine antenatal care, 81.2% of the women reported having their blood pressure taken, 65.8% had urine samples collected, 61.1% had blood samples taken, 73.9% listened to the baby's heartbeat, and 62.5% reported assessing danger signs. A majority of respondents (90.1%) reported logistical challenges as their reason for not using ANC, and 87.3% mentioned perceived inefficiency or lack of necessity. The majority (97.1%) of the women received fewer than 90 tablets of iron folate.

When we compared ANC attendance among the three arms, the gain-framed arm had lower attendance (85.7%) than both the control arm (86%) and the loss-framed arm (91.8%). In terms of when the first ANC visit occurred, 68.5% of the participants in the gain-framed arm, 83.3% in the loss-framed arm, and 79.9% in the control arm started before 16 weeks. In terms of ANC frequency, 81.5% of the gain-framed arms, 95% of the loss-framed arms, and 87.6% of the control arms had limited ANC (≤ 4 times).

Regarding reasons for ANC nonattendance, logistical barriers were high across arms: gain-framed (96.4%), loss-framed (87.5%), and control (85.2%). Resource limitations were another challenge, with the gain-framed arm at 82.1%, the loss-framed arm at 81.3%, and the control arm at 88.9%. Conversely, perceived ineffectiveness was high for the loss-framed arm (93.8%) and control arm (92.6%) (Table 3).

**Table 3:** Experiences in prenatal care practices, Jimma Zone, Oromia Regional State, South West Ethiopia (*n* = 588)

Variables	Categories	Gain framed n(%)	Loss framed n(%)	Control n(%)	Total n(%)	Statistical test (*P*.Value)
Parity	Primiparous	3(1.5)	7(3.6)	19(10)	29(4.9)	χ2 = 15.10 *p* = 0.001
	Multiparous	193(98.5)	189(96.4)	177(90)	559(95.1)	
Pregnancy outcome	Live birth	182(92.9)	182(92.9)	185(94)	549(93.4)	χ2 = 5.53 *p* = 0.478
	Stillbirth	8(4.1)	9(4.6)	4(20)	21(3.6)	
	Miscarriage	5(2.6)	4(2)	3(2)	12(2)	
	Abortion	1(0.5)	1(0.5)	4(2)	6(1)	
Receive ANC^a^ at least once	Yes	168(85.7)	180(91.8)	169(86)	517(87.9)	χ2 = 2.85 *p* = 0.241
	No	28(14.3)	16(8.2)	27(14)	71(12.1)	
Place of ANC follow-up	Health post	12(7.1)	7(3.9)	3(1.8)	22(4.3)	χ2 = 62.71 *p* < 0.001
	Health center	135(80.4)	84(46.7)	110(65.1)	329(63.6)	
	Hospital	21(12.5)	86(47.8)	54(32)	161(31.1)	
	Other places	0(0)	3(1.7)	2(1.2)	5(1)	
Time at first antenatal care	< = 16 weeks	115(68.5)	150(83.3)	135(79.9)	400(77.4)	χ2 = 11.97 *p* = 0.003
	> 16 weeks	53(31.5)	30(16.7)	34(20.1)	117(22.6)	
Antenatal care frequency	Limited ANC(< = 4times)	137(81.5)	171(95)	148(87.6)	456(88.2)	χ2 = 15.32 *p* < 0.001
	Regular ANC (> 4times)	31(18.5)	9(5)	21(12.4)	61(11.8(	
Counseling During ANC,	Delivery Preparation	118(70.2)	106(58.9)	138(81.7)	362(70)	χ2 = 23.21 *p* < 0.001
	Breastfeeding	102(60.7)	92(51.1)	134(79.3)	328(63.4)	
	Child spacing	90(53.6)	68(37.8)	133(78.7)	291(56.3)	
	Immunization	115(68.5)	89(49.4)	116(68.6)	320(61.9)	
	Danger sign	107(63.7)	93(51.7)	110(65.1)	310(60)	
	Nutrition	86(51.2)	104(57.8)	114(67.5)	304(58.8)	
TT^b^ Vaccination	Yes	149(88.7)	153(85)	122(72.2)	424(82)	χ2 = 16.95 *p* < 0.001
	No	19(11.3)	27(15)	47(27.8)	93(18)	
TT injection frequency	One	19(12.8)	6(3.9)	21(17.2)	46(10.8)	χ2 = 27.96 *p* < 0.001
	Two	80(53.7)	61(39.9)	39(32)	180(42.5)	
	Three or more	50(33.6)	86(56.2)	62(50.8)	198(46.7)	
LLITN^c^ Use	Always	65(33.2)	42(21.4)	78(39.8)	185(31.5)	χ2 = 59.83 *p* < 0.001
	Sometimes	43(21.9)	37(18.9)	57(29.1)	137(23.3)	
	Rarely	21(10.7)	18(9.2)	33(16.8)	72(12.2)	
	Never	67(34.2)	99(50.5)	28(14.3)	194(33)	
Experience during the last antenatal care visits	BP^d^ measurement	135(80.4)	144(80)	141(83.4)	420(81.2)	χ2 = 17.74 *p* = 0.025
	Urine sample	105(62.5)	92(51.1)	143(84.6)	340(65.8)	
	Blood sample	103(61.3)	86(47.8)	127(75.1)	316(61.1)	
	Check baby's heartbeat	131(78)	130(72.2)	121(71.6)	382(73.9)	
	Check vaginal bleeding	105(62.5)	103(57.2)	115(68)	323(62.5)	
Reasons for Not using antenatal care	Logistical barrier	27(96.4)	14(87.5)	23(85.2)	64(90.1)	χ2 = 7.65 *p* = 0.106
	Resource limitation	23(82.1)	13(81.3)	24(88.9)	60(84.5)	
	Perceived Ineffectiveness	22(78.6)	15(93.8)	25(92.6)	62(87.3)	
Iron tablet received	< 90 Tablets	128(98.5)	153(96.8)	119(96)	400(97.1)	χ2 = 1.45 *p* = 0.484
	> 90 Tablets	2(1.5)	5(3.2)	5(4)	12(2.9)	

^a^*ANC* Antenatal care

^b^*TT* Tetanus toxoid

^c^*LLITN* Long lasting Integrated Treated Nets

^d^*BP* Blood Pressure

### Experience in intrapartum care

Most (74.7%) CI (73.2, 79.9) births occurred in a health facility. The majority of births (97.1%) were vaginal, with 1.5% and 1.4% born via cesarean section (CS) and forceps/vacuum extraction, respectively. The top three reasons women chose not to give birth in a health institution were financial challenges (94.0%), personal/informational barriers (84.6%), and logistical barriers (92.6%). The majority (95.4%) of respondents reported that they prepared for birth, mainly by saving money. Less than half (30.1%) of the women experienced serious health problems during pregnancy, labor, or birth, with severe bleeding (11.9%) and severe headaches (9.2%) being the most commonly reported issues.

Health centers were the preferred location for deliveries, with the gain-framed arm at 65.3%, the loss-framed arm at 62.8%, and the control arm at 71.4%. The gain-framed arm had a greater percentage of home deliveries (31.6%) than did the control arm (17.3%) and loss-framed arm (27%). Logistic barriers to giving birth at health facilities were more prominent in the gain-framed arm (89.3%) than in the loss-framed arm (67.7%) and control arm (72%). Informational barriers were greater in the control arm (33.1%) than in the gain-framed arm (15%) and the loss-framed arm (16.8%). Birth preparedness was greater in the control arm (79.6%) than in the gain-framed arm (61.7%) and the loss-framed arm (74%). Serious health problems during pregnancy, labor, or birth were comparable across arms; 25% of the participants were in the gain-framed arm, 32.7% in the loss-framed arm, and 32.1%in the control arm. Unweighted births were more prevalent in the gain-framed arm (51%) than in the loss-framed arm (40.3%) and control arm (36.2%) (Table 4).

**Table 4:** Experiences in intrapartum care, Jimma Zone, Oromia Regional State, Southwest Ethiopia (*n* = 588)

Variables	Category	Gain framed n(%)	Loss framed n(%)	Control n(%)	Total n(%)	Statistical test (*P*.Value)
Birth location	Health facility ^a^	134(68.4)	143(73)	162(82.7)	439(74.7)	χ2 = 11.02 *p* = 0.004
	Home	62(31.6)	53(27)	34(17.3)	149(25.3)	
Delivery Assistance	HCP**	143(73)	145(73.9)	163(83.2)	451(76.7)	χ2 = 1.45 *p* = 0.484
	TBA	32(16.3)	8(4.1)	3(1.5)	43(7.3)	
	Family members	21(10.7)	43(21.9)	30(15.3)	94(16)	
Delivery method	CS	2(1)	4(2)	3(1.5)	9(1.5)	χ2 = 0.94 *p* = 0.919
	Forceps/vacuum	2(1)	3(1.5)	3(1.5)	8(1.4)	
	Vaginal delivery	192(98)	189(96.4)	190(96.9)	571(97.1)	
Non Facility Birth Reasons	Logistic barrier	46(89.3)	47(67.7)	45(17)	138(92.6)	χ2 = 8.66 *p* = 0.07
	Financial Barriers:	43(18.9)	48(15.8)	49(22.5)	140(94)	
	Personal Barriers	39(6.5)	42(16.8)	45(33.3)	126(84.6)	
Birth Preparedness	Yes	121(61.7)	144(74)	154(79.6)	419(95.4)	χ2 = 13.55 *p* = 0.001
	No	13(6.6)	1(0.5)	6(3.1)	20(4.6)	
Birth preparedness type	Save money	126(64.3)	143(73)	150(76.5)	419(95.4)	χ2 = 61.35 *p* < 0.001
	Organize transport	18(9.2)	16(8.2)	40(20.4)	74(16.9)	
	Identity skilled attendant	8(4.1)	4(2)	35(17.9)	47(10.7)	
	Identify blood donors	2(1)	0(0)	22(11.2)	24(5.5)	
Pregnancy complication	Yes	49(25)	64(32.7)	64(32.1)	177(30.1)	χ2 = 3.49 *p* = 0.175
	No	147(75)	132(67.3)	132(67.3)	411(69.9)	
Types of Pregnancy complication	Severe bleeding	27(13.8)	33(16.8)	10(5.1)	70(11.9)	χ2 = 36.42 *p* < 0.001
	Severe headache	7(3.6)	22(11.2)	25(12.8)	54(9.2)	
	Blurred vision	5(2.6)	5(2.6)	13(6.6)	23(3.9)	
	High fever	1(0.5)	2(1)	5(2.6)	8(1.4)	
	Labor > 12 h	8(4.1)	0(0)	7(3.6)	15(2.6)	
	Others^c^	1(0.5)	2(1)	3(1.5)	6(1)	

^a^(7 health post, 391 health center, and 41 hospital)

^b^(41 doctor, 393 nurse/midwife, and 17 health extension workers)

^*^(3 retained placenta, 2 failure to progress and 1 placental complication)

^**^Healthcare Provider

### Experience in postpartum care

Most (60.4%) of the respondents reported having received postnatal check-ups. Regarding the length of stay in the health facility for postnatal care (PNC), approximately half (51.5%) stayed less than 24 h. The majority (81.1%) of the participants received postnatal check-ups within the first 24 h. This study showed that only 28% of those who gave birth at home received postnatal check-ups. The most noted postnatal services mentioned by women were; taking temperature (43.0%), recognizing the baby's urgent needs (36.2%), observing breastfeeding (39.1%), measuring blood pressure (42.3%), assessing vaginal bleeding (43.4%), and family planning (42.3%).

By comparing postnatal care checks along the three arms, we found that the gain-framed arm had the highest proportion (64.8%), followed by the loss-framed arm (58.7%) and the control arm (57.7%). Regarding the timing of postnatal checks, immediate postnatal checks were notably more frequent in the gain-framed arm (30.7%) than in the loss-framed (8.7%) and control (13.3%) arms (Table 5).

**Table 5:** Experiences in postnatal care, Jimma Zone, Oromia Regional State, Southwest Ethiopia (*n* = 588)

Variables	Category	Gain framed n(%)	Loss framed n(%)	Control n(%)	Total n(%)	Statistical test (*P*.Value)
PNC^a^_Checkup	Yes	127(64.8)	115(58.7)	113(57.7)	355(60.4)	χ2 = 2.83 *p* = 0.243
	No	69(35.2)	81(41.3)	83(42.3)	233(39.6)	
PNC_Length of Stay	< 24 h	36(18.4)	108(55.1)	82(40.7)	226(51.5)	χ2 = 38.65 *p* < 0.001
	> 24 h	98(49.9)	34(17.3)	81(41.3)	213(48.5)	
PNC_Checkup Time	Immediate Check	39(30.7)	10(8.7)	15(13.3)	64(18)	χ2 = 18.97 *p* < 0.05
	Within the first 24 h	88(69.3)	105(91.3)	95(84.1)	288(81.1)	
	Beyond 24 h	0(0)	0(0)	3(2.7)	3(0.8)	
PNC_Checkup Type	Measure temperature	82(41.8)	44(22.4)	127(64.8)	253(43)	χ2 = 12.59 *p* = 0.981
	Baby's urgent needs	79(40.3)	36(18.4)	98(50)	213(36.2)	
	Observe breastfeeding	75(38.3)	42(21.4)	113(57.7)	230(39.1)	
	Check blood pressure	84(42.9)	43(21.9)	122(62.2)	249(42.3)	
	Assess vaginal bleeding	87(44.4)	42(21.4)	126(64.3)	255(43.4)	
	Family planning	81(41.3)	45(23)	123(62.8)	249(42.3)	
PNC_Counseling	Maternal Danger Signs	96(49)	60(30.6)	92(46.9)	248(42.2)	χ2 = 15.51 *p* = 0.595
	Newborn Danger Signs	97(49.5)	58(29.6)	92(46.9)	247(42)	
	Newborn warmth	94(48)	49(25)	90(45.9)	233(39.6)	
	Exclusive breastfeeding	95(48.5)	58(29.6)	91(46.4)	244(41.5)	
	Child Immunization	91(46.4)	45(23)	89(45.4)	225(38.3)	

^a^*PNC* Postnatal Care

### Experiences with messages on maternal health

Most (66%) study participants reported that they had heard of maternal health (MH) messages. Traditional media was the primary source of health messages for approximately 63.8% of the study participants, followed by healthcare professionals (46.9%), community health workers (33.2%), family members (24.3%), friends (24.7%), and other sources (23%). Regarding exposure frequency, 31.5% reported frequent exposure, 16.2% sometimes, 16.2% occasionally, 32.5%, rarely, and 19.9% never. Approximately 29.6% of respondents actively sought information, 37.4% relied heavily on incoming sources, and 33% utilized both approaches.

In comparing the three arms regarding maternal health message exposure and information-seeking behavior, similar exposure rates were observed, with 67.9% in the gain-framed group, 65.8% in the loss-framed group, and 64.3% in the control group. Conventional media was the primary source across all arms, reaching 67.9%, 70.9%, and 52.6% in the gain-framed, loss-framed, and control arms, respectively. The frequency of message exposure varied significantly, with 42.9% in the gain-framed group, 18.9% in the loss-framed group, and 32.7% in the control group reporting frequent exposure. Information-seeking behavior showed disparities, as 41.3% of participants in the gain-framed group actively sought information, whereas 28.6% of those in the loss-framed group and 18.9% of those in the control group did (Table 6).

**Table 6:** Exposure to maternal health messages, Jimma Zone, Oromia Regional State, Southwest Ethiopia (*n* = 588)

Variables	Categories	Gain framed n(%)	Loss framed n(%)	Control n(%)	Total n(%)	Statistical test (*P*.Value)
Exposed to MH^a^ messages	Yes	133(67.9)	129(65.8)	126(64.3)	388(66)	χ2 = 0.56 *p* = 0.756
	No	63(32.1)	67(34.2)	70(35.7)	200(34)	
Source of health messages	Healthcare professionals	97(49.5)	90(45.9)	89(45.4)	276(46.9)	χ2 = 26.46 *p* = 0.023
	Family members	51(26)	49(25)	43(21.9)	143(24.3)	
	Friends	54(27.6)	48(24.5)	43(21.9)	145(24.7)	
	Community health workers	77(39.3)	63(32.1)	55(28.1)	195(33.2)	
	Traditional media^a^	133(67.9)	139(70.9)	103(52.6)	375(63.8)	
	Other sources^b^	56(28.6)	35(17.9)	44(22.4)	135(23)	
Message frequency	Frequently	84(42.9)	37(18.9)	64(32.7)	185(31.5)	χ2 = 49.85 *p* < 0.005
	Occasionally	19(9.7)	26(13.3)	50(25.5)	95(16.2)	
	Rarely	61(31.1)	83(42.3)	47(24)	191(32.5)	
	Never	32(16.3)	50(25.5)	35(17.9)	117(19.9)	
Information seeking behavior	Actively seeking	81(41.3)	56(28.6)	37(18.9)	174(29.6)	χ2 = 28.19 *p* < 0.005
	Incoming information	54(27.6)	84(42.9)	82(41.8)	220(37.4)	
	Both	61(31.1)	56(28.6)	77(39.3)	194(33)	

^a^(206 radio, 134 television, 35 newspaper)

^b^(39 digital platform, 78 meetings, 18 religious leaders)

### Factors associated with maternal health service use

Twenty-three variables were independently entered into the bivariable logistic regression analysis using an enter method, and 11 of them demonstrated an association with the dependent variable at a *p*-value < 0.25. Variables that were associated with the dependent variable at the bivariate level were simultaneously adjusted in binary logistic regression. The educational level of pregnant women, wealth category, means of transportation, knowledge of maternal healthcare, self-efficacy in maternal healthcare, and exposure to maternal health messages didn’t show significant association with the dependent variable. On the other hand, proximity to the health facility, transportation cost, perceived respectful care, privacy during procedures, and attitude toward maternal health service showed significant association.

Multivariable logistic regression results showed that women with immediate access were significantly more likely to utilize maternal health services. They had 6.6 times higher odds of using these services compared to those with challenging access [AOR = 6.6, 95% CI: 2.39, 18.16], with a highly significant *p*-value of less than 0.001. This indicates that proximity to health facilities greatly increases the likelihood of maternal health service utilization. Women with moderate access had 2.2 times higher odds of utilizing maternal health services compared to those with challenging access [AOR = 2.2, 95% CI: 1.00, 5.32], with a marginally significant *p*-value of 0.08. Although the relationship is not as strong as for immediate access, moderate proximity still positively influences service utilization. Reasonable access was also positively associated with maternal health service utilization. Women in this category were 2.6 times more likely to use maternal health services compared to those with challenging access [AOR = 2.6, 95% CI: 1.10, 6.27], with a significant *p*-value of 0.02.

Women who did not experience economic hardship were three times more likely to utilize maternal health services than those who did [AOR = 3, (95% CI: 1.97, 4.61)], with a highly significant relationship (*p* < 0.001). Women who received respectful care were 2.3 times more likely to use maternal health services compared with those who reported non-respectful care [AOR = 2.3, (95% CI: 1.07, 5.11)], with a significant relationship (*p* = 0.03). Women who received private care had 2.4 times higher odds of utilizing maternal health services compared to those who did not have private care [AOR = 2.4, 95% CI: 1.47, 4.06], with a highly significant *p*-value of less than 0.001. This indicates the importance of privacy in healthcare settings to promote service use.

Women with a favorable attitude towards maternal healthcare were 2.2 times more likely to utilize maternal health services compared to those with an unfavorable attitude [AOR = 2.2, 95% CI: 1.48, 3.24], with a highly significant *p*-value of less than 0.001. A positive attitude towards healthcare is thus a significant motivator for service utilization (Table 7).

**Table 7:** Predictors of maternal health service utilization, Jimma, Oromia Regional State, Southwest Ethiopia

Variables	Categories	Maternal Health Service Utilization		Odds Ratio (95%CI)		*P* value (Adjusted)
		Yes n(%)	No n(%)	Crude	Adjusted	
Access to Health Facility^a^	Immediate access	17(5.3)	42(15.7)	9(3.41,23.49)	6.6(2.39, 18.16)	< .001
	Moderate access	114(35.6)	67(25)	2.1(0.92,4.93)	2.2(1.00,5.32)	.08
	Reasonable access	133(41.6)	103(38.4)	2.8(1.23,6.40)	2.6(1.10,6.27)	.02
	Limited access	27(8.4)	48(17.9)	6.4(2.58,16.07)	6.5(2.46,17.22)	< .001
	Challenging access	29 (9.1)	8(3)	1	1	
Economic hardship	Yes	129(40.3)	54(20.1)	1	1	
	No	191(59.7)	214(79.9)	2.7(1.84,3.89)	3(1.97,4.61)	< .001
Perceived respectful care	Yes	12(3.8)	28(10.4)	3(1.49,6.01)	2.3(1.07,5.11)	.03
	No	308(96.3)	240(89.6)	1	1	
Privacy of care	Private	31(9.7)	66(24.6)	0.3(0.21,0.52)	2.4(1.47,4.06)	< .001
	Not Private	289(90.3)	202(75.4)	1	1	
Attitude toward Maternal Health	Favorable attitude	121(37.8)	126(47)	1.5(1.05,2.03)	2.2(1.48,3.24)	< .001
	Unfavorable attitude	199(62.2)	142(53)	1	1	

^a^Immediate access (0–15 min), moderate access (15–30 min), reasonable access (30–60 min), limited access (60–90 min), and challenging access (90 + minutes)

## Discussion

The utilization of maternal health services plays a crucial role in decreasing maternal mortality rates. Research has indicated that improved utilization of maternal health services, such as receiving care from skilled attendants during childbirth, is strongly linked to improved maternal survival rates [41–43].

According to the current study, the overall maternal health service utilization was 54.4%, 87.9% of mothers attended antenatal care, 74.7% used institutional delivery, and 60.4% used postnatal care. Higher utilization rates are observed for antenatal care compared with the global average (81%), whereas institutional delivery and postnatal care show lower utilization rates compared with the global average (81%) [44]. Additionally, a pooled analysis of demographic and health surveys across 32 sub-Saharan African countries revealed varying levels of skilled antenatal care service utilization, ranging from 99.2% in Gambia to 8.4% in Burundi [45]. Furthermore, a study in Ethiopia showed that 62% of pregnant women received antenatal care, 48% opted for institutional delivery, and 34% received postnatal care [46]. The data suggest a relatively high utilization of maternal health services, low early ANC utilization, and a high service dropout rate in the Jimma Zone, which could be attributed to challenges related to accessibility, geographical disparities, infrastructure limitations, or sociocultural factors influencing healthcare-seeking behavior.

The result from the complementary study showed that 79.9% of the respondents can read mobile text messages, while only 54.1% of them can send them. This finding is comparable with a study conducted in India, where only 52.5% of the participants could type and send text messages [47]. However, it is lower than the finding of study conducted in northern Ethiopia, where 91% and 87.3% of participants could read and send mobile text messages, respectively [48]. The difference might be due to variations in educational status of study participants. Although only about half of the participants in the study were able to send a text message, four out of five could read them, which would make a mobile phone-based messaging intervention feasible. The complementary study also showed that nearly all respondents (98.9%) preferred receiving health information in Afan Oromo via text messages, underscoring the importance of language and cultural relevance in mobile health interventions. The preference for receiving message in the common language of their locality was also reported by the majority of participants in other studies [47, 48]. The findings suggests the widespread accessibility and acceptance of mobile technology among pregnant women in Jimma Zone.

Addressing challenges such as network issues and costs is crucial for maximizing the effectiveness of mobile health interventions. In contexts where network reliability can be a barrier, employing alternative communication methods such as volunteers and health extension workers could significantly enhance the effectiveness of the intervention. These strategies can facilitate direct communication with pregnant women in local communities, ensuring that health information reaches those who may have limited access to mobile networks or digital literacy. Volunteers and health extension workers can also provide personalized support, answer questions, and reinforce health messages in culturally appropriate ways, thereby enhancing engagement and adherence to health advice. Integrating these additional support mechanisms into existing mobile health strategies not only addresses infrastructural challenges but also strengthens community-based healthcare delivery, ultimately improving maternal and child health outcomes.

The current study revealed that proximity to health facilities was an independent predictor of maternal health service utilization; hence, women with immediate access, reasonable access, moderate, and limited access were more likely to utilize maternal health services than women with challenging access. This result aligns with a study in Nepal which found that geographical barriers significantly affected the utilization of maternal health services, with women living in mountainous and remote areas less likely to access antenatal care and skilled birth attendance compared to those in more accessible areas [49], a study in India, which showed that distance to health facilities was a major determinant of whether women opted for institutional deliveries, with those residing in rural areas facing greater challenges in accessing these services [50], findings from a systematic review on maternal care barriers in African low-income countries [51], a study in sub-Saharan Africa [52], a study in Tanzania which showed that women residing more than five kilometers from a healthcare facility were notably less likely to attend four or more antenatal care visits [53], findings from Kenya, where proximity to healthcare facilities was one of the strongest predictors of maternal health service utilization [54]; these studies highlighted the significant impact of access on the utilization of maternal health services. This suggests that as the distance to health facility increases, the likelihood of women utilizing maternal health services decreases. It also stresses the critical role of addressing geographical inequities and enhancing access to health facilities to improve maternal health service use.

Limited financial resources can affect the ability to access maternal health services. The current study revealed that women without a financial burden demonstrated greater odds of using maternal health services than those who experienced economic hardship. This finding aligns with earlier studies that have recognized economic hardships as a major obstacle in obtaining maternal healthcare in low- and middle-income nations [51, 55], a study in Bangladesh, found that financial constraints significantly influenced the decision to seek maternal health services, with many women unable to afford the costs associated with healthcare visits, medications, and necessary tests [56], a study in India, found that financial limitations were a critical barrier to maternal health service utilization. Women from poorer households were less likely to access institutional deliveries and postnatal care due to the costs associated with these services [57], a study in Uganda highlighted that economic hardship was a major factor affecting maternal health service utilization [58], and research in Pakistan also demonstrated that financial barriers were a major obstacle to accessing maternal health services [59]. Improving access to maternal healthcare services requires addressing these barriers and empowering women through education and equitable healthcare access. This challenge could be attributed to male dominance and a lack of autonomous decision-making among the study population. Overcoming these obstacles and ensuring equal access to healthcare are crucial steps in enhancing maternal healthcare services.

Respectful care was another significant factor in maternal health service use. Based on the results of the current study conducted in the Jimma Zone, women who experienced respectful care had greater odds of utilizing maternal health services. This finding supports a study conducted by Sheferaw ED [60], where the absence of respectful maternity care was recognized as restrictive to the utilization of maternity services, another study conducted by Kawish AB [61] also showed that the provision of respectful care plays a crucial role in promoting health-seeking behaviors among pregnant women. A study in Ethiopia found that respectful maternity care, including effective communication, emotional support, and maintaining the dignity of the mother, significantly increased the likelihood of women seeking skilled birth attendance and postnatal care [62]. In Tanzania, research revealed that women who experienced disrespectful care, including being scolded or ignored by healthcare providers, were less likely to return to health facilities for postnatal care or future deliveries [63]. A study in Kenya identified disrespectful and abusive care during childbirth as a major barrier to maternal health service utilization, with instances of verbal abuse, physical mistreatment, and lack of informed consent, discouraging women from seeking care at health facilities for subsequent pregnancies [64]. Similarly, a study In Latin America, Mexico reported that women who felt respected and valued during their interactions with healthcare providers were more likely to attend antenatal care and deliver in health facilities [65]. The absence of respectful care in the area may have contributed to a lack of discharge accountability by healthcare providers. Therefore, ensuring a dignified and respectful working environment could contribute to an increase in health facility delivery and a reduction in maternal mortality.

Maintaining privacy and confidentiality during examinations and procedures is one of the 12 domains of respectful maternity care [66]. According to the current study, women who reported private procedures had 2.4 times greater odds of using maternal health services. This finding is supported by, another study in Ethiopia, which found that women who perceived their consultations and procedures as private and confidential were more likely to seek skilled birth attendance and complete their postnatal care regimen [67]. In Nepal maintaining privacy during maternity care was a key factor influencing women's decisions to seek institutional delivery and postnatal care [68]. Similarly, a study in Brazil showed that women who experienced respectful care with maintained privacy during childbirth had higher odds of utilizing maternal health services [69]. In Nigeria women who reported that their privacy was maintained during childbirth were more likely to return for postnatal care and recommend institutional delivery to others [70], A cross-sectional study conducted in the Kingdom of Saudi Arabia that explored the occurrence of respect perceived by women giving birth and its effect on maternal health service utilization [71]. This study highlights the importance of ensuring privacy and confidentiality in healthcare services, particularly in maternity care.

Finally, women with a favorable attitude toward maternal healthcare demonstrated greater odds of using maternal health services. Similarly, a study in rural Ethiopia revealed that women's attitudes toward maternal health services were significantly associated with their intentions to use maternal health services [72]. In Bangladesh, women’s attitudes toward maternal health services played a crucial role in their utilization, with positive attitudes were associated with increased attendance at antenatal care visits and higher rates of institutional deliveries [73]. A study in Kenya found that women with positive attitudes toward maternal health services were more likely to utilize antenatal, delivery, and postnatal care. Health education programs aimed at improve perceptions of maternal health services were shown to be effective in increasing service utilization [74]. Poor attitudes toward maternal health services might have been due to the low literacy rate in the area and previous negative experiences. This underscores the importance of addressing factors that that shape women`s attitudes toward maternal healthcare.

## Limitation of the study

This study has some limitations that should be considered when interpreting its findings. Gestational age was determined based on the last menstrual period (LMP) and fundal height, which may be inaccurate due to irregular menstrual periods, oligohydramnios, or polyhydramnios. Additionally, the research did not investigate maternal–fetal diseases and abnormal placental transplantation, potentially limiting the comprehensive understanding of maternal health outcomes. Another limitation is the focus on pregnant mothers or pregnant mother’s families who own a mobile phone, which may overlook those without access to mobile technology.

## Conclusion

Although antenatal care attendance was high, early initiation of antenatal care was low with high dropout rates. Most births occurred at healthcare facilities, and postnatal service utilization was relatively high; however, some mothers gave birth at home and did not attend postnatal care. Factors significantly affecting maternal health service utilization included access to health facilities, economic hardship, perceived respectful care, privacy, and attitudes toward maternal health.

A knowledge gap in maternal health care was evident, with many participants lacking sufficient information. Additionally, a large percentage of pregnant mothers had unfavorable attitudes and low self-efficacy regarding maternity care. Conventional media was the primary source of maternal health information, highlighting the need for tailored communication strategies. Addressing barriers such as distance, transportation costs, respectful care, privacy, knowledge gaps, attitudes, and self-efficacy is crucial for improving maternal healthcare services, requiring tailored interventions to meet the unique needs of diverse groups.

Future research should include an assessment of materno-fetal characteristics and consider studies in which all pregnant women are provided with mobile phones, which would minimize selection bias and allow for a more equitable assessment of maternal health care across socioeconomic groups.

## Data Availability

No datasets were generated or analysed during the current study.

## Abbreviations

ANC: Antenatal care
ANC1: First antenatal care visit
ANC4: Fourth antenatal care visit
CI: Confidence interval
CS: Cesarean section
DE: Design effect
GA: Gestational age
ICC: Interclass correlation
KASE: Knowledge, attitude and self-efficacy
LLITN: Long lasting integrated treated nets
LMP: Last menstrual period
MH: Maternal health
MMR: Maternal mortality ratio
PHCU: Primary health care unit
PNC: Postnatal care
SDG: Sustainable development goal
SPSS: Software package for social science
TBA: Traditional birth attendance
TT: Tetanus toxoid
WHO: World health organization
